# Resveratrol as a Therapeutic Agent for Alzheimer's Disease

**DOI:** 10.1155/2014/350516

**Published:** 2014-11-26

**Authors:** Teng Ma, Meng-Shan Tan, Jin-Tai Yu, Lan Tan

**Affiliations:** ^1^Department of Neurology, Qingdao Hiser Hospital, School of Medicine, Qingdao University, Qingdao 266034, China; ^2^Department of Neurology, Qingdao Municipal Hospital, School of Medicine, Qingdao University, No. 5 Donghai Middle Road, Qingdao 266071, China; ^3^Department of Neurology, Qingdao Municipal Hospital, College of Medicine and Pharmaceutics, Ocean University of China, Qingdao 266003, China

## Abstract

Alzheimer's disease (AD) is the most common cause of dementia, but there is no effective therapy till now. The pathogenic mechanisms of AD are considerably complex, including A*β* accumulation, tau protein phosphorylation, oxidative stress, and inflammation. Exactly, resveratrol, a polyphenol in red wine and many plants, is indicated to show the neuroprotective effect on mechanisms mostly above. Recent years, there are numerous researches about resveratrol acting on AD in many models, both in vitro and in vivo. However, the effects of resveratrol are limited by its pool bioavailability; therefore researchers have been trying a variety of methods to improve the efficiency. This review summarizes the recent studies in cell cultures and animal models, mainly discusses the molecular mechanisms of the neuroprotective effects of resveratrol, and thus investigates the therapeutic potential in AD.

## 1. Introduction

AD is a progressive, degenerative disorder and by far the most common cause of dementia. The pathogenesis and progression of AD are not well understood; however, the characteristic histopathologic features are studied extensively, including neuritic plaques (senile plaques), neurofibrillary tangles, loss of neurons and synaptic connections, and glial proliferations [1]. Although the detailed causes of AD are still in debates, two pathological hallmarks have been widely identified: senile plaques (SPs) and neurofibrillary tangles (NFTs). SPs consist of deposits of *β*-amyloid protein (A*β*) as a core which is surrounded by dystrophic neuritis, activated astrocytes, and microglia. The NTFs are formed by hyperphosphorylation and abnormal deposition of tau proteins [2]. Scientists have been trying for a long time to treat and to alleviate the cognitive impairment. Unfortunately, no currently available treatment has been shown to reverse existing deficits or to prevent disease progression [3].

Many pharmacotherapies have been applied clinically to ameliorate the symptoms of AD, such as several cholinesterase inhibitors and memantine, an N-methyl-D-aspartate- (NMDA-) type glutamate receptor antagonist drug. However, these drugs have been shown to produce diverse side effects and yield relatively modest benefits, by reason of the complex mechanisms [4]. For example, cholinesterase inhibitors present only temporary and modest effects on improvement of memory impairment and motor function, but the side effects tend to be intolerable, such as nausea and diarrhea. Tacrine, another cholinesterase inhibitor, has been limited in clinical treatment because of the poor oral bioavailability and severe hepatotoxicity. Memantine, which used to be a hopeful drug, has proved to be less effective clinically than the cholinesterase inhibitors [5]. Therefore, there is an urgent need for new strategies based on multiple pathomechanisms of AD.

Resveratrol (3, 5, 4′-trihydroxy-*trans*-stilbene) is a kind of polyphenol produced in several plants, especially grapes skin and seeds, and a phytoalexin against pathogens such as bacteria or fungi [6]. An epidemiological research was reported between moderate red wine consumption and a low incidence of cardiovascular disease, which was termed “French Paradox” [7]. Afterwards the studies indicated that resveratrol showed the diverse biological activities, such as antioxidant, anti-inflammatory, phytoestrogenic, vasorelaxing, cardioprotective, and anticarcinogenic, which help it to play important roles in treatment against cardiovascular diseases and cancers, as well as degenerative disorders in brain, including AD [8–10]. Numerous researches have been carried out to find whether resveratrol can take a therapeutic potential in AD and other neurodegenerative diseases, including various models in vitro and in vivo. Although the researches on resveratrol and AD are still in the infancy and the long-term effects of clinical supplementation in human are not known, more and more studies have indicated that resveratrol was involved in several pathophysiologic courses of AD [8]. In this review, we searched the MEDLINE, EMBASE, CINAHL, and BIOSIS previews up to January, 2014, with the terms “resveratrol” or “trans-resveratrol” or “RES” or “RSV” and “Alzheimer's disease” or “Alzheimer disease” or “AD.” The search terms were used to identify relevant therapeutic effects of resveratrol on AD, both in vitro and in vivo, as well as clinical trials. On the whole, 97 publications were included in the full-text screening. We also enumerate the roles of resveratrol in AD pathogenesis and in AD therapy and discuss the benefit of resveratrol as a therapeutic agent of AD.

## 2. Complex Pathogenesis and Multiple Targets for Treatment

**Figure 1:**
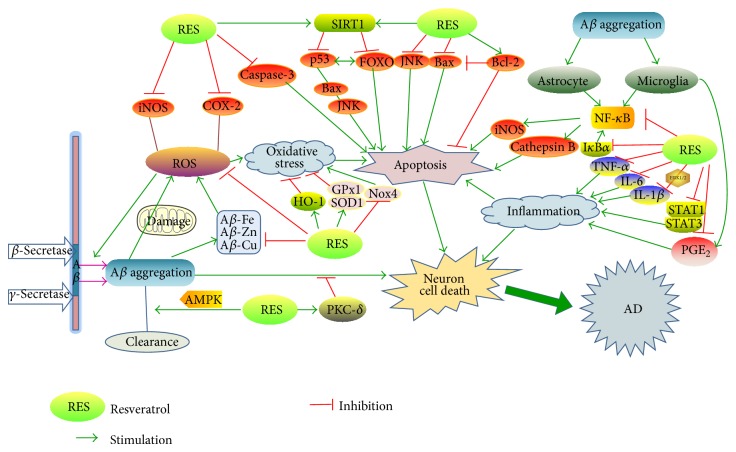
Neuroprotective functions of resveratrol in AD pathogenesis. (1) APP is cleaved by *β*- and *γ*-secretases and the production of A*β* aggregates together. (2) Resveratrol promotes the intracellular clearance of A*β* without influencing the generation of A*β*, by activating AMPK independently of SIRT1. (3) The phosphorylation of PKC, mainly PKC-*δ*, is induced by resveratrol and consequently plays a major role in the neuroprotective properties against A*β*-induced toxicity. (4) Resveratrol reduces the generations of A*β*-Fe, A*β*-Cu, and A*β*-Zn and thus reduces their toxicity. (5) ROS is produced by damaged mitochondria during oxidative stress, mainly iNOS and COX-2, and plays an important role in apoptosis. Resveratrol reduces iNOS and COX-2 levels and increases the production of HO-1 to attenuate oxidative damage. (6) Resveratrol decreases the expression of the ROS-producing enzyme Nox4 but increases the expression of ROS-inactivating enzymes, SOD1 and GPx1. (7) Resveratrol influences the A*β*-induced apoptotic signaling pathway, including restoring the decrease of Bcl-XL expression, inhibiting the expression of Bax, blocking the activation of JNK, and suppressing the increase of NF-*κ*B DNA binding. (8) Resveratrol can inhibit PGE_2_ formation by activated microglial cells. (9) A*β* aggregation is responsible for the activation of astrocytes and microglia, which excrete cytokines, such as IL-1*β*, IL-6, and TNF-*α*, which all were transcriptionally controlled by NF-*κ*B. Resveratrol inhibits the increase of STAT1, STAT3, and I*κ*B*α* phosphorylation.

The pathogenic mechanisms of AD are considerably complex (Figure 1), so that most of the treatments are unable to take every aspect into account. AD is defined by characteristic pathologic features, especially neuritic plaques and NFTs, which are clearly visible by microscopy in brains of those afflicted by AD [11]. Neuritic plaques, also called senile plaques, are extracellular insoluble deposits of A*β* peptide. In 1992, the amyloid hypothesis postulated that A*β* deposits were the elementary cause of AD and since then the hypothesis had been gradually acknowledged [12]. Amyloid precursor protein (APP) is abnormally cleaved by *β*- and *γ*-secretases, but not *α*-secretase, which leads to excessive extracellular accumulation of A*β* in the cortex and hippocampus in AD brains. A*β* accumulation leads to the progressive loss of neurons, interdict of neural circuits, and neurological decline characteristic of AD [13]. However, APP will be cleaved into soluble amyloid precursor *α* (sAPP*α*) by *α*-secretase pathway, which can relatively reduce the generation of A*β* and protect neurons by promoting axon growth. Therefore, *β*- and *γ*-secretases are considered to be the pivotal targets of AD pharmaceuticals, but on the contrary enhancement of *α*-secretase may produce the similar effect in anti-AD treatment.

Oxidative stress has been strongly involved in the pathogenesis of neurodegenerative diseases including AD. The brain tends to be very prone to oxidative imbalance and vulnerable to oxidative damage, due to the higher levels of polyunsaturated fatty acids and the relative lack of antioxidant systems, compared with other organs [5, 14]. It is regarded as an underlying cause of AD because of the reactive oxygen species (ROS) and reactive nitrogen species during the early development of the disease, preceding the formation of senile plaques [5]. A large number of ROS are produced by damaged mitochondria during oxidative stress, mainly including inducing nitric oxide synthase (iNOS) and cyclooxygenase-2 (COX-2) and these productions may damage the mitochondrial and cellular proteins and nucleic acids, causing lipid peroxidation and resulting in the loss of membrane integrity [15]. ROS increasing A*β* production and A*β* inducing oxidative stress, a vicious circle between ROS and A*β* accumulation, may accelerate the progression of AD [6]. Thus, antioxidants have been demonstrated to protect against A*β*-induced neurotoxicity [5]. Resveratrol, as an antioxidant, is found to reduce iNOS levels and alleviate lipid peroxidation in neuron cells and, however, increase the production of heme oxygenase-1 (HO-1) to attenuate oxidative damage.

Recent evidences suggest that neuroinflammation is an important contributor to pathogenesis of AD. Inflammatory changes, including the activation of microglia, astrocytes, and macrophages, are observed in the AD brain, particularly in the amyloid deposits [16]. Numerous evidences in cell cultures and mouse models indicate that aggregated A*β* is responsible for the activation of astrocytes and microglia [17], causing the release of large amounts of proinflammatory mediators, including cytokines, free radicals, and nitric oxide (NO), all of which increase the generation of insoluble A*β* [18, 19]. A*β* triggers the respiratory burst of microglia and produces ROS and tumor necrosis factor alpha (TNF-*α*), which aggravates A*β* deposition and further neuronal dysfunction and eventual death [20]. The potentially significant contribution of inflammatory mechanisms in AD has prompted consideration of anti-inflammatory treatment strategies [5].

Tau protein is a highly soluble microtubule-associated protein (MAP). These proteins mostly exist in neurons, both central and peripheral nervous systems, and perform the function of stabilizing microtubules [21]. Tau is a phosphoprotein and the phosphorylation of tau is regulated by many kinases. When the tau protein is phosphorylated, it deviates from microtubules and aggregates abnormally, which is known as tauopathies, including AD [22]. The phosphorylated tau protein results in the disassembling of microtubules and aggregates to form neurofibrillary tangles, a hallmark of AD pathology. Once polymerized into neurofibrillary tangles, the tau loses the function of connection to tubulin and microtubule assembly. Thus, inhibition of pathological hyperphosphorylation of tau may be a therapeutic target for AD and other tauopathies [23, 24].

Neuron-synapse loss was considered as one of the main features of AD and other neurodegenerative disorders and possibly the direct cause of dementia occurrence and deterioration [25, 26]. Synaptic dysfunction is found in the early phase of AD, and loss of synapses appears in the later phase. As a result, the excitatory transmission in hippocampus and cerebral cortex is inhibited, which contributes to memory loss [27, 28]. Although A*β* deposition may cause neuron loss, the main way of neuron-synapse loss is apoptosis. In AD models, there are various factors which can launch the apoptosis, such as oxidative stress, glucose metabolism disorder, and excitotoxic mitochondrial damage. In addition, some substrates, for example, p53, FOXO, and ROS, are involved in the apoptosis process of AD. Thus, downregulation of apoptosis seems to be a potential treatment of AD.

The treatments of many progressive neurodegenerative maladies, such as Huntington's disease, Parkinson's disease, and AD, have been the puzzles in neurology. Resveratrol is known to have beneficial metabolic effects and is considered a mimic of caloric restriction [29]. In recent years, resveratrol has been shown to have neuroprotective effects for its pleiotropic functions. Researches indicate that resveratrol may promote clearance of A*β*, mitigate oxidative stress, decrease the production of proinflammatory factors, scavenge free radicals, inhibit platelet aggregation, suppress activation of astrocytes and microglia, and reduce neuron cell death [9]. In all, studies show that resveratrol possesses potent neuroprotective properties in several models, both in vitro and in vivo.

## 3. Neuroprotective Effects of Resveratrol In Vivo

Resveratrol has been found to exhibit neuroprotective function in animal models, mostly rat models (Table 1). It is reported that daily moderate consumption of the red wine Cabernet Sauvignon significantly reduced AD-type amyloid neuropathology and attenuated A*β*-associated spatial memory deterioration in the Tg2576 mouse model. However, the role of resveratrol in this study was not clear because the content of resveratrol in Cabernet Sauvignon was much lower than the minimal effective concentration shown to promote A*β* clearance in vitro [30]. Recent studies about Tg2576 mice indicated that extracellular accumulation of soluble A*β* oligomers was largely responsible for AD dementia and memory deficits [31]. Grape seed polyphenolic extract (GPSE) treatment reduced oligomerization of A*β* peptide and attenuated amyloid-associated cognitive impairments in Tg2576 mice [32]. In resveratrol-treated APP/PS1 mice, there was a significant reduction in the number of activated microglia, suggesting that resveratrol decreased inflammation, at least in part, independently of its effect on amyloid deposition [17].

In male C67BL/6J mice, resveratrol reduced serum TNF-*α* and macrophage infiltration of adipose tissue, attenuated neuroinflammation and oxidative stress in the hippocampus, and enhances cognitive function in HFD-fed mice [33]. In another study on male C57Bl/6 mice, resveratrol was provided in the food (150 *μ*g resveratrol/gram food). As a result, resveratrol-treated mice were improved in spatial orientation and memory performance, which was paralleled by an increased microvascular density in the hippocampus and decreased number of vacuolar abnormalities in both hippocampal and cortical microvascular endothelial cells [10]. Moreover, in C57Bl/6J mice model, research showed that in resveratrol-treated high fat-fed mice, there was a significant decrease in ambulatory locomotor activity as well as a tendency to decrease the number of rears. The data demonstrated that the effects of resveratrol were seen in both muscle and brown adipose tissue, and this might result in an increase in mitochondrial function, which translated into an increase in energy expenditure, an improved aerobic capacity, and an enhanced sensorimotor function [34].

To extend their in vitro results, Dasgupta and Milbrandt conducted an experiment and found that intraperitoneal injection of resveratrol acutely activated AMP-activated kinase (AMPK) in the brain, probably due to the phosphorylation and inhibition of acetyl-CoA carboxylase (ACC) [35]. Streptozotocin is usually used to build diabetes rat models, which may show up impairment of memory. In intracerebroventricular (ICV) injection of streptozotocin (STZ) model rats, there could be impairment in learning and memory in addition to decreased choline acetyltransferase levels in the hippocampus, because of the prolonged impairment of brain glucose and energy metabolism. Sharma and Gupta indicated that ICV STZ group rats chronically treated with trans-resveratrol showed significantly increased retention latencies and shorter transfer latencies on the elevated plus maze, but no significant difference in the locomotor activity of sham [36]. Other rat models were made by ICV administration of colchicine (15 *μ*g/5 *μ*L), which could induce impaired cognitive functions. Chronically treated with resveratrol for 25 days, the malondialdehyde (MDA) and nitrite levels reduced, the glutathione and acetylcholinesterase activity recovered, and the cognitive impairment induced by colchicine was greatly improved [37].

In the adult male Wistar rats with intraperitoneal injection of 55 mg/kg streptozotocin, treatment with resveratrol significantly prevented the increase in AChE activity, especially in the cerebral cortex and hippocampus. In addition, treatment with resveratrol was able to prevent the increase in AChE activity and consequently in cognitive impairment in diabetic rats, which meant that this polyphenol could modulate cholinergic neurotransmission and consequently improve cognition [38]. Furthermore, another study showed oral resveratrol induced the expression of brain derived neurotrophic factor (BDNF) mRNA in the hippocampus of rat brain in the male Sprague-Dawley rat models. BDNF had several neuroprotective roles similar to those of resveratrol and the study indicated resveratrol might have positive effects on the expression of BDNF [39].

The silent information regulator 2 (SIR2) gene promotes longevity in a variety of organisms and may underlie the health benefits of caloric restriction. SIRT1, one of the seven mammalian homologues of the sirtuin family of NAD^+^-dependent deacetylases, has recently been demonstrated to contribute to cellular regulation and take part in several signal transduction pathways [56]. It is found that resveratrol possesses the ability to activate SIRT1, although there is a viewpoint that resveratrol is not a direct activator [57]. In the inducible p25 transgenic mouse, a model of AD and tauopathies, ICV injection of resveratrol resulted in the activation of SIRT1. After 5 weeks of p25 induction, cell death and neurodegeneration were obvious in the hippocampus of the vehicle treated animals, but administration of resveratrol could reduce neurodegeneration in CA1 and CA3 regions of the hippocampus because of the lower levels of the apoptotic marker-activated caspase-3 and glial fibrillary acidic protein (GFAP), a marker of astrogliosis. p25-green fluorescent protein (GFP) expressing neurons were more robust in the hippocampus of resveratrol-treated rats, demonstrating the neurons might tolerate and survive p25 expression. Learning capability improved after treatment with resveratrol for 3 weeks. That is to say resveratrol provides neuroprotection and prevents cognitive decline in animal models [40].

Therefore, resveratrol is beneficial for animal neurodegenerative disorders induced by some neurotoxicity. The possible mechanisms may be responsible for its antioxidant and anti-inflammatory properties. Pretreatment with resveratrol significantly attenuates oxidative stress damage and improves motor and cognitive impairment [58, 59].

## 4. Neuroprotective Functions of Resveratrol In Vitro

The neuroprotective effects of resveratrol have been investigated in several in vitro models, either rat cells or human cells (Table 2). In hippocampal slices prepared from 10-day-old Sprague-Dawley rat pups, glutamate rapidly induced monocyte chemotactic protein-1 (MCP-1) production in the hippocampus. Resveratrol downregulated glutamate-induced extracellular signal-regulated kinase (ERK) activation and then resulted in decreased interleukin-1*β* (IL-1*β*) expression and the subsequent downregulation of MCP-1 in the hippocampus [41]. In the rat cortical primary neurons, a decrease in peroxisome proliferator-activated receptor-gamma coactivator- (PGC-) 1*α* was observed, suggesting that SIRT1 activity was increased following resveratrol treatment of primary neurons. Furthermore, increased levels of SIRT1 in primary neurons played protective roles against neurotoxicity induced by p25 or mutant SOD1. Resveratrol provided neuroprotection and prevented cognitive decline through the deacetylation of p53 and the sequent decreasing of p53 level, an important mediator of cell death [40]. A study on primary cortical neuron cultures showed that resveratrol was effective in protecting against NMDA-induced neuronal death by inhibiting the elevation of intracellular calcium and production of ROS [42]. In a study on primary hippocampal cultured cells, A*β*_25–35_ was used to establish A*β*-induced neuronal cell death, because of its similar mechanism of toxic effect. A pretreatment of hippocampal neuronal cells with resveratrol (15–40 *μ*M) significantly reduced A*β*_25–35_-induced cell death in a dose-dependent manner, with a maximal effect obtained at 25 *μ*M. Meanwhile, cotreatment and posttreatment with resveratrol showed similar neuroprotective effects, although the effects seemed to be with a somewhat lower potency. The phosphorylation of protein kinase C (PKC) was induced by resveratrol in a dose-dependent manner, with maximal effects seen at 20–30 *μ*M. Moreover, resveratrol slightly decreased the phosphorylation of PKC-d, but did not affect the phosphorylation of PKC-*α*/*β*II, PKC-*μ* (Ser916), and PKC-*θ* (Thr538), suggesting that PKC-*δ* (Thr505) was involved in the neuroprotective effects of resveratrol. In short, the PKC pathway played a major role in the neuroprotective-neurorescuing properties of resveratrol against A*β*-induced toxicity in hippocampal neurons [43]. In mixed (glial/neuronal) hippocampal cultured cells, treatment with the NO donor SNP (100 *μ*M) resulted in cell damage and resveratrol (5 ± 25 *μ*M) treatment increases cell survival, and this protective effect was significant at 5 *μ*M and maximal at the highest (25 *μ*M) concentration tested. Thereby, resveratrol, as well as other red wine polyphenols, was able to rescue hippocampal cells against toxicity induced by NO, possibly mediated by antioxidant activities. Interestingly, in this experiment resveratrol and catechin, but not quercetin, did not involve inhibitory effects on intracellular enzymes such as COX/LOX, NOS, and PKC. However, other researches found that resveratrol (10 *μ*M) inhibited NO generation and suppressed iNOS in lipopolysaccharide- (LPS-) activated macrophages. The contradiction may be due to different experimental conditions, including cell types and toxic agents [44]. Resveratrol can also inhibit prostaglandin E_2_ (PGE_2_) and free radical formation by activated microglial cells in primary cultured microglial cells, because of the modulation of multiple events in the COX/PGE_2_ pathway. Resveratrol can reduce LPS-mediated expression of microsomal prostaglandin E synthase-1 (mPGES-1) and COX-1, but not COX-2 expression [45]. In cultured rat astroglioma C6 cells, A*β* induced a time-dependent reduction of cell growth, but pretreatment with resveratrol protected the cells from the toxicity of A*β*. In this research, resveratrol inhibited NO production and iNOS expression in response to A*β* in a concentration-dependent manner. Furthermore, resveratrol showed the inhibitory effects on the accumulation of PGE_2_ by A*β* through downregulation of COX-2 in C6 cells. In addition, pretreatment with resveratrol prevented the translocation of NF-*κ*B by A*β* [46].

A recent study was executed on murine macrophage cell line RAW 264.7, murine microglial cell line BV-2, and murine bone marrow-derived pro-B cell line Ba/F3. It was found that resveratrol treatment significantly reduced multiple cytokines in LPS-stimulated RAW 264.7 and BV-2 cells, such as IL-6, M-CSF, CD54, IL-1ra, and TNF-*α*, which were all transcriptionally controlled by NF-*κ*B. NF-*κ*B signaling controls the expression of iNOS and cathespin B, which are the known toxic factors involved in apoptosis. Resveratrol decreased the levels of phosphorylated IKK*α*, I*κ*B*α*, and NF-*κ*B in RAW 264.7 and BV-2 cells and even greatly inhibited the phosphorylation of Akt, a kinase controlled by MyD88 upon Toll-like receptor (TLR) 4 activation. On the other hand, resveratrol also inhibited signal transducer and activator of transcription (STAT) 1 and STAT3 activation by LPS in these two cell lines and reduced the expression of iNOS and COX-2 in a dose-dependent manner. Resveratrol treatment significantly lowered the levels of FLAG-tagged TLR4 coimmunoprecipitating with GFP-tagged TLR4, which meant resveratrol obstructed TLR4 oligomerization upon LPS stimulation. Furthermore, resveratrol dose dependently inhibited the increase of STAT1, STAT3, and I*κ*B*α* phosphorylation, as well as TNF-*α* and IL-6 secretion, induced by fibrillar A*β*. Therefore, resveratrol inhibited the microglial inflammatory responses triggered by both LPS and A*β* [17]. Resveratrol was reported to exert a protective effect against cytotoxicity induced by A*β*_25–35_ or A*β*_1–42_ in cultured rat pheochromocytoma (PC12) cells. Resveratrol was also found to inhibit A*β*_25–35_ induced apoptotic cell death with the exhibition of morphological alterations and increase of TUNEL-positive cells. In this study, resveratrol was indicated to inhibit the A*β*_25–35_-induced dissipation of the mitochondrial membrane potential and intracellular ROI accumulation. Moreover, resveratrol was reported to influence the A*β*_25–35_-induced apoptotic signaling pathway, including preventing the cleavage of PARP, rescuing the decrease of Bcl-XL expression, inhibiting the expression of the proapoptotic Bax protein, blocking the activation of JNK via phosphorylation, and suppressing the increase of NF-*κ*B DNA binding [47]. It was also found in rat PC12 cells that resveratrol remodels A*β* soluble oligomers and fibrillar conformers into large nontoxic aggregates in a dose-dependent manner [48]. Melatonin (N-acetyl-5-methoxy-tryptamine, MEL) was reported to be one of the potential candidates involved in antioxidant and neuroprotection in AD. In cultured murine HT22 hippocampal cells and primary hippocampal neuron cells, cotreatment with MEL and resveratrol was more effective in preventing A*β*_1–42_-induced neurotoxicity. MEL and resveratrol inhibited the activation of ERK, reduced ROS production, rescued glutathione (GSH) levels, and attenuated neuronal cell death. The A*β*_1–42_-induced increase of GSK3*β* activity and activation of AMPK were inhibited by either MEL or resveratrol alone, and cotreatment of these two compounds exerted a synergistic effect [49].

Other than these rat cells as in vitro models, there are many human cell lines applied to investigate the protective effects of resveratrol in AD (Table 2). For example, HEK293 cell, a specific cell line originally derived from human embryonic kidney cells grown in tissue culture, transfected with human APP_695_ was used to find the efficacy of resveratrol on A*β* clearance. The research found resveratrol had no effect on *α*-, *β*-, or *γ*-secretase-mediated cleavages of APP, which meant resveratrol did not influence the APP metabolism and A*β* production. Resveratrol did not facilitate A*β* degradation by neutral endopeptidase (NEP), endothelin-converting enzyme- (ECE-) 1 and ECE-2, or insulin-degrading enzyme (IDE) in HEK293 cells but promoted a proteasome-dependent intracellular clearance of A*β* without increasing total proteasome activity [50]. In addition to its own antioxidant effect, resveratrol also regulated the gene expression of prooxidative and antioxidative enzymes in human umbilical vein endothelial cells (HUVEC). In HUVEC-derived EA.hy926 cells, resveratrol was found to decrease the expression of the ROS-producing enzyme Nox4 but increase the expression of ROS-inactivating enzymes, SOD1 and GPx1. By these results, resveratrol displayed a novel approach to reduce endothelial oxidative stress [51]. Resveratrol could directly bind to both monomeric and fibrillar amyloid structures in human hippocampus slice, so resveratrol could directly stain A*β* plaques [60]. SH-SY5Y neuroblastoma cells were applied to investigate the inhibitory function of resveratrol on beta-amyloid oligomeric cytotoxicity. It was indicated that resveratrol played protective roles in AD through suppressing the extension of amyloidogenic A*β* peptides and disaggregating A*β*42 fibrils but not inhibiting A*β*42 oligomer formation [52]. SH-SY5Y human neuroblastoma cell cultures treated with A*β* complexes in presence or absence of resveratrol were applied to find the roles of resveratrol in A*β* metabolism. It was indicated that resveratrol had no direct antiamyloidogenic and fibril-destibilizing effects but mainly played a scavenging role through its neuroprotective activity against A*β* as well as A*β*-metal complexes. Resveratrol, a ROS scavenger, reduced the generations of A*β*-Fe, A*β*-Cu, and A*β*-Zn and thus reduced their toxicity. However, resveratrol was not sufficient to fully block A*β*-Al and A*β*-Cu toxicity because of the possibly different pathways, which were not only oxidative stress dependent [2]. Studies are performed to investigate the effect of resveratrol on the production of prostanoids induced by IL-1*β* in SK-N-SH cells, a human neuroblastoma cell line. It was demonstrated that PGE_2_ and PGD_2_ production was drastically reduced by resveratrol, even at very low doses. However, the same dose of resveratrol did not greatly reduce mPGES-1 and COX-2 immunoreactivities, as well as COX-1. That was to say resveratrol reduced prostanoid synthesis and free radical generation without interfering with the expression of COX-1, COX-2, or mPGES-1 but via the reduction of COX-2 activity [53]. Another study was carried out to find the role of resveratrol in preventing cell death due to oxidative stress in SK-N-BE, another neuroblastoma cell line. Resveratrol exerted antioxidant effects against H_2_O_2_ and 6-OHDA, and the neuroprotective mechanism was sensitive to sirtinol, indicating an involvement of SIRT1 enzymatic activation. Resveratrol inhibited the toxicity induced by TAT-*α*-syn (A30P) protein aggregation and A*β*42 fibrils, both increasing ROS production. In addition, resveratrol SIRT1 independently reduced A*β*42 toxicity, influenced A*β*42 fibril production and steadiness, and reduced intracellular A*β*42-dependent ROS generation [54]. Although resveratrol is a potent activator of SIRT1 and the metabolic functions are chiefly through the deacetylase activity of SIRT1, resveratrol can also play a neuroprotective role by activating AMPK independently of SIRT1. Resveratrol reduced A*β* accumulation by activating AMPK signaling in HEK293 (APP-HEK293) and N2a (APP-N2a) cell lines and in primary neuronal cultures. Resveratrol activated AMPK via increasing cytosolic calcium levels and expediting CaMKK*β*-dependent phosphorylation. Resveratrol reduced A*β* aggregation by activating autophagy and by promoting the lysosomal degradation of A*β* [55].

## 5. Resveratrol as a Therapeutic Agent for Other Neurodegenerative Diseases

Because of the neuroprotective effects of resveratrol mentioned above, the polyphenol has been found to play an important role in neurodegenerative diseases other than Alzheimer's disease, for example, Parkinson's disease (PD) and amyotrophic lateral sclerosis (ALS). The beneficial effects of resveratrol are not only antioxidant and anti-inflammatory actions but also activation of SIRT1 and vitagenes, which can prevent the deleterious effects triggered by oxidative stress. Recently, researches showed that resveratrol may offer a promising approach for treatment of these neurodegenerative disorders.

PD is a neuronal degeneration of dopaminergic neurons located in substantia nigra pars compacta (SNpc) and the PD patients often show increasing muscle rigidity, resting tremors, bradykinesia, and, in extreme cases, a nearly complete loss of movement. Ferretta et al. performed an in vitro experiment to find the effect of resveratrol treatment on primary fibroblast cultures from two patients with early-onset PD linked to different Park2 mutations. The results showed that resveratrol regulates energy homeostasis via activation of AMPK and SIRT1 and increase in mRNA expression of a number of PGC-1*α*'s target genes resulting in increased mitochondrial oxidative function [61]. Lin et al. use an in vitro rotenone-induced PD model to verify the neuroprotective effects of resveratrol. Resveratrol acted in a neuroprotective manner to increase both HO-1 expression and autophagic flux with no effect on cell viability. Moreover, the effects of a pharmacological inducer of HO-1 were similar to those of resveratrol and protected against rotenone-induced cell death [62]. In the study of Khan et al., male Wistar rats were used to make 6-OHDA-induced PD rat model, which were pretreated with resveratrol and subjected to unilateral intrastriatal injection of 6-OHDA. As a consequence, resveratrol was found to decrease the level of thiobarbituric acid reactive substances (TBARS), upregulate the antioxidant status, and lower the dopamine loss. In addition, resveratrol has been found to suppress COX-2 expression [63]. Another research by Lofrumento et al. was performed to analyze the neuroprotective effects of resveratrol in 1-methyl-4-phenyl-1,2,3,6-tetrahydropyridine (MPTP-) induced PD mouse model. Results showed that resveratrol mitigated glial activation and decreased the levels of IL-1*β*, IL-6, and TNF-*α*, as well as their respective receptors in the SNpc of MPTP-treated mice [64]. Furthermore, it was reported that inhibition of AMPK caused suppression of SIRT1 activity and reduced protective effects of resveratrol on rotenone-induced apoptosis, which meant AMPK-SIRT1-autophagy pathway played an important role in the neuroprotection by resveratrol on PD cellular models [65]. These findings suggest that the resveratrol is an attractive alternative in the treatment of PD.

ALS is an adult onset, incurable fatal neurodegenerative disease, characterized by the selective loss of motor neurons in brain, brainstem, and spinal cord. Song et al. used SOD1 (G93A) transgenic mice, a classic animal model of ALS, to test the neuroprotective effects of resveratrol. In this research, resveratrol was found to regulate the expression of Sirt1 and PGC1-*α* and improve the lipid peroxidation, inhibiting p53 and its downstream apoptotic pathway. As a result, resveratrol significantly delayed the disease onset, extended the lifespan in the mice, ameliorated motor neuron loss, and alleviated the atrophy and mitochondrial dysfunction in the muscle fibers. That is to say the antioxidant and antiapoptotic effects of resveratrol are the major beneficial roles against ALS [66]. Another research by Mancuso et al., also about the effects of resveratrol in SOD1 (G93A) ALS mice, showed that the protective effects of resveratrol were associated with increased expression and activation of sirtuin 1 and AMPK in the ventral spinal cord. Both mediators improved normalization of the autophagic flux and, more importantly, increased mitochondrial biogenesis in the SOD1 (G93A) spinal cord [67]. Similar study carried out by Wang et al. proved the upregulation of the expression of SIRT1, which protected the ALS cell model from mutant SOD1-mediated toxicity [68]. All of the studies represented a potential therapeutic target for preventing the motor neuron degeneration in ALS patients.

## 6. Poor Bioavailability of Resveratrol and Potential Solutions

In spite of the high bioactivity of resveratrol, we are still puzzled by its low bioavailability. As we mentioned before, resveratrol plays a very important role in Alzheimer's disease, especially in vitro. However, it is difficult to show the same effects when expanded to in vivo animal models and human clinical trials. To date, there have been different results about the anti-inflammatory properties of resveratrol, among which some conclusions seemed to be conflicting or controversial. Because of the poor bioavailability of resveratrol, the concentrations of resveratrol at target tissues and cells appear far from sufficient to demonstrate efficacy in humans. Studies have shown that the oral absorption of resveratrol appeared to be at least 75%; however the bioavailability was poor on account of the rapid and extensive metabolism [69]. Resveratrol, whose half-life is only 8–14 minutes, has been found to be quickly metabolized into sulfate and glucuronide metabolites in liver and intestinal epithelial cells in human [70, 71]. The poor bioavailability of resveratrol is also associated with the poor aqueous solubility, which is reported as <1 mg/mL [72]. Additionally, trans-resveratrol is photosensitive, is easily oxidized, and presents unfavorable pharmacokinetics [73]. Therefore, successful clinical application of resveratrol is a severe challenge for the medical as well as pharmaceutical technology. Researchers have tried different approaches to improve the solubility and bioavailability, including coadministration of inhibitors of trans-resveratrol metabolism, searching for analogs and elaboration of new trans-resveratrol delivery systems [74].

In recent years, a number of researches have focused on novel formulation approaches to stabilize and preserve resveratrol from degradation and increase its solubility so as to improve its bioavailability, to achieve a sustained release, and ultimately to transport resveratrol to specific locations via multiparticulate forms and colloidal carriers. Methylated resveratrol analogs exert similar biological activities that are comparable with those of the resveratrol. However, the methylated resveratrol analogs manifest better bioavailability as they are more easily transported into the cell and more resistant to degradation. Kang et al. firstly reported an artificial biosynthetic pathway to obtain methylated resveratrol compounds in the* E. coli* culture joined with resveratrol biosynthetic genes and two resveratrol O-methyltransferase genes [75]. Amiot et al. developed an innovative soluble galenic form, which consisted of natural trans-resveratrol powder (40 mg) dissolved in a complex mixture containing polysorbate 20 and polyglyceryl-3-dioleate. Consequently, the novel formulation significantly improved the oral absorption of trans-resveratrol and the total trans-resveratrol bioavailability (+780%). A single dose in two lipid caplets (40 mg) of soluble trans-resveratrol was capable to enhance trans-resveratrol plasma concentrations around 10-fold, compared to the dry powder form [74]. Amri et al. applied monodisperse functionalized porous polymeric microspheres for the sake of the stabilization and preservation of resveratrol [76]. As another formulation, the complexation of trans-resveratrol with *β*-cyclodextrin (*β*-CD), hydroxypropyl-*β*-CD (HP-*β*-CD), randomly methylated-*β*-cyclodextrin (RM-*β*-CD), and maltosyl-*β*-cyclodextrin (G2-*β*-CD) has been investigated to increase trans-resveratrol solubility. On the other hand, either multiparticulate forms in the millimeter to micrometer range or colloidal carriers in the nanometer range have been used in several studies as the formulations to achieve targeted and/or sustained release of resveratrol. Das et al. found that Ca-pectinate beads and Zn-pectinate beads could encapsulate a large amount of resveratrol (>97.5%) and could be used for delayed release and site-specific delivery to the lower gastrointestinal tract [77, 78]. In the research of Frozza et al., trans-resveratrol was loaded into lipid-core nanocapsules. As a result, trans-resveratrol-loaded lipid-core nanocapsules increased the concentration of trans-resveratrol in the brain tissue and gastrointestinal safety was improved when compared with free trans-resveratrol [73].

Microparticulate systems may be a good choice to control the release and improve bioavailability of resveratrol. Recently, researchers incorporated resveratrol into cross-linked chitosan microspheres by vanillin to improve the stabilization, and the encapsulation efficiency of resveratrol in microspheres was up to 93.68% [79]. In another study, microparticles were produced by crosslinking pectin molecules with zinc ions and with glutaraldehyde as hardening agent for pectins, as a specific delivery to colon with >94% encapsulation efficiency [80]. Cyclodextrins can solubilize hydrophobic drugs in pharmaceutical applications and crosslink to form polymers used for drug delivery. By means of the transformation of crystalline cyclodextrins into amorphous mixtures of isomeric derivatives, the bioavailability of poorly soluble drugs, including resveratrol, can be improved and the aqueous solubility enhanced. In a research of resveratrol in human trial, the antioxidant effect was enhanced by treatment with the *β*-cyclodextrin- (*β*CD-) containing formulation compared to formulation containing trans-resveratrol alone [81]. Resveratrol nanosuspensions were performed by high pressure homogenization and the stable nanosuspensions resulted in the enhancement of solubility of the drug improving its overall bioavailability [82]. Solid lipid nanoparticles (SLN) may be an alternative which were used as a carrier for resveratrol in many studies. Resveratrol solubility, stability, and intracellular delivery were all increased by loading into SLN [83]. In the study of Jose et al., resveratrol-loaded SLN were equally effective as free resveratrol and SLN could remarkably increase the brain concentration of resveratrol as compared to free resveratrol. Therefore the results showed that the resveratrol-loaded SLN serve as promising therapeutic systems in brain tissue [84].

Another novel method to deliver encapsulated drug is vesicular system, which means the colloidal particles containing concentric bilayer, capable of transporting both hydrophilic and hydrophobic drugs. Vesicular systems could increase bioavailability and provide therapeutic activity for a longer duration, including liposomes, transferosomes, phytosomes, and ethosomes. In a study, liposome formulations were chosen to optimize the loading of the rigid hydrophobic resveratrol and the liposomes showed the protection of resveratrol against biological degradation and metabolism thus improving the drug bioavailability [85]. Liposomal resveratrol formulations designed for intravenous administration have also shown interesting properties [76]. Different surfactants and lipids were used for the preparation of transferosomes and ethosomes and were characterized for size, zeta potential, stability, and permeation. Pangeni et al. used Span 60 and Span 80 to prepare resveratrol-loaded niosomes. As a result, the latter seemed to be more stable with narrow particle size distribution and high entrapment efficiency proving the improvement in bioavailability [86]. Nanosponges are emerging nanohorizon in drug delivery through nanotechnology. Owing to their small size and porous nature they can combine with poorly soluble resveratrol within the matrix and improve the bioavailability [87]. Encapsulating resveratrol in *β*-CD nanosponges improved its solubility in water and researchers believed that resveratrol-loaded nanosponges were viable for oral and topical delivery systems [88]. In a recent study, Pund and his colleagues formulated a lipid-based delivery system of resveratrol with self-nanoemulsifying ability, containing Acrysol K 150 as a lipid and mixture of Labrasol and Transcutol HP as the surfactant system. As a result, lipid based nanoemulsifying resveratrol enhanced solubility in vitro and therapeutic efficacy in vivo [89].

## 7. Clinical Researches of Resveratrol in AD

There is growing evidence that resveratrol may benefit in AD treatment, both in vitro and in vivo. However, there is not a complete large-scale clinical trial coming to a conclusion until now. Resveratrol seems to be well tolerated and no marked toxicity was reported. Bioavailability of resveratrol is one of the focal points because the compound is poorly bioavailable, low water soluble, and chemically unstable [69, 90]. After oral administration or intravenous injection of resveratrol in humans, resveratrol was rapidly metabolized within 2 hours, even a peak in 30 minutes [71]. Several studies in vivo in animals and humans demonstrated a very low intestinal uptake of resveratrol, and it was difficult to detect unmetabolized resveratrol in the circulating plasma [69]. It was indicated that trans-resveratrol pharmacokinetics following oral administration of 200 mg thrice daily was independent of age. However, the plasma concentrations of trans-resveratrol were relatively low [91]. Although in vivo evidence is emerging in animal models that resveratrol is bioavailable and bioactive [55], conclusive results in human trials are still lacking. In recent years many researchers have tried a variety of ways to find the effective administration methods of resveratrol [74, 92–94], but none of them is fully satisfied. As selective ways, some researches put emphasis up on the resveratrol metabolites responsible for its biological activity and the analogs with similar neuroprotective properties [9].

There are currently numerous clinical trials to investigate the effects of resveratrol on neurodegenerative diseases, of course including AD, in spite of the enormous difficulties [29]. In a double-blind, placebo-controlled, crossover investigation on healthy young volunteers consuming 500 mg resveratrol, levels of total hemoglobin were remarkably higher in the frontal cortex during task performance [95]. However, cognitive performance was not affected [29, 96]. In a randomized controlled trial (RCT) versus placebo, 60 AD patients were given liquid resveratrol with glucose and malate as a dietary supplement delivered in grape juice. The ADAS-cog scores were assessed at regular intervals up to 1 year after study commencement [96, 97]. In another multi-interventional clinical trial in mild cognitive impairment (MCI), resveratrol supplementation or caloric restriction or omega-3 supplementation or placebo was provided for 6 months, and then the ADAS-cog scores would be assessed [97].

## 8. Conclusion

Resveratrol is a novel agent for treatment of AD because of its multiple mechanisms in neuroprotection. As the treatment of AD is still a worldwide problem, therapeutic potential of resveratrol has attracted the interest of researchers to shift emphasis on. Plenty of trials have been exerted to find the concrete details of the neuroprotective mechanisms of resveratrol in cell cultures and animal models of AD. For the antioxidative and anti-inflammatory functions, resveratrol truly represents the beneficial effects on AD. Furthermore, some official systematic clinical trials about resveratrol treatment in AD have also been underway. Although the difficulties of clinical application are enormous, such as the bioavailability, dosage, and side effects, scientists are still trying to seek out the detailed mechanism and the suitable clinical administration of resveratrol.

## Figures and Tables

**Table 1:** Neuroprotective effects of resveratrol in vivo.

Animals	Administration	Effects	Reference
Tg2576 mice	Drinking Cabernet Sauvignon	Reduced amyloid neuropathology and attenuated spatial memory deterioration.	[30]

Tg2576 mice	Fed with GPSE	Reduced oligomerization of A*β* peptide and attenuated cognitive impairments.	[32]

APP/PS1 mice	Diet with resveratrol	Reduced the number of activated microglia.	[17]

C67BL/6J mice	Diet with trans-resveratrol daily	Reduced serum TNF-*α* and enhanced cognitive function.	[33]

C57Bl/6 mice	Resveratrol in food	(1) Increased microvascular density and decreased number of vacuolar abnormalities. (2) Improved in spatial orientation and memory performance.	[10]

C57Bl/6J mice	Resveratrol in food	Increased mitochondrial function, improved aerobic capacity, and enhanced sensorimotor function.	[34]

2-month-old male mice	i.p. injection of resveratrol	Activated AMPK in the brain.	[35]

Wistar rats, ICV injection of STZ	ICV injection of resveratrol	Increased retention latencies and shorter transfer latencies.	[36]

Rats, ICV administration of colchicine	Chronically treated with resveratrol (p.o.)	MDA levels reduced but GSH and AchE activity recovered.	[37]

Wistar rats, i.p. injection of STZ	i.p. injection of resveratrol	Modulates cholinergic neurotransmission and consequently improves cognition.	[38]

Sprague-Dawley rat	Oral resveratrol	Resveratrol may have positive effects on the expression of BDNF.	[39]

Inducible p25 transgenic mice	ICV injection of resveratrol	Reduces neurodegeneration in hippocampus and prevents cognitive decline.	[40]

**Table 2:** Neuroprotective effects of resveratrol in vitro.

Cell	Exposure	Effects of resveratrol	Reference
Hippocampal slices	Glutamate treated	Downregulated ERK activation, decreased IL-1*β* expression, and downregulated MCP-1 in the hippocampus.	[41]

Rat cortical primary neurons	Treatment with ionomycin and H_2_O_2_	Increased SIRT1 activity and prevented cognitive decline.	[40]

Primary cortical neurons	Exposure to NMDA	Inhibited the elevation of intracellular calcium and production of ROS.	[42]

Primary hippocampal cells	A*β*_25–35_ induced	Reduced A*β*_25–35_-induced cell death and decreased the phosphorylation of PKC-*δ*.	[43]

Mixed (glial/neuronal) hippocampal cells	Treated with SNP or SIN-1	Rescued hippocampal cells against NO-induced toxicity and inhibited NO generation and suppressed iNOS in LPS-activated macrophages.	[44]

Primary microglial cells	LPS-induced	Inhibit PGE_2_ and free radical formation and reduce LPS-mediated expression of mPGES-1 and COX-1.	[45]

Rat astroglioma C6 cells	Treated with A*β*	Reduced NO production and iNOS expression, inhibited accumulation of PGE_2_, downregulated COX-2 expression, and prevented the translocation of NF-*κ*B.	[46]

RAW 264.7, BV-2, and Ba/F3 cells	Stimulated with LPS	Reduced multiple cytokines, decreased the levels of phosphorylated IKK*α*, I*κ*B*α*, and NF-*κ*B, inhibited STAT1 and STAT3 activation, and reduced the expression of iNOS and COX-2.	[17]

PC12 cells	A*β*_25–35_ or A*β*_1–42_ induced	Restored the decrease of Bcl-XL expression, inhibited the expression of Bax, blocked the activation of JNK, and suppressed the increase of NF-*κ*B DNA binding.	[47]

PC12 cells	Treated with A*β*	Remodels A*β* soluble oligomers and fibrillar conformers into large nontoxic aggregates.	[48]

Murine HT22 hippocampal cells and primary hippocampal neuron cells	Treated with A*β*, MEL, and resveratrol	MEL and resveratrol inhibited the activation of ERK, reduced ROS production, rescued GSH levels, and attenuated neuronal cell death. Cotreatment exerted a synergistic effect.	[49]

APP_695_-HEK293 cell	Treated with A*β*	Did not influence the APP metabolism and A*β* production but promoted a proteasome-dependent intracellular clearance of A*β*.	[50]

HUVEC-derived EA.hy926 cells	DMNQ-induced	Decreased the expression of Nox4 but increased the expression of SOD1 and GPx1.	[51]

SH-SY5Y neuroblastoma cells	A*β* induced	Suppressed the extension of amyloidogenic A*β* peptides and disaggregated A*β*42 fibrils.	[52]

SH-SY5Y neuroblastoma cells	Treated with A*β* complexes	Reduced the generations of A*β*-Fe, A*β*-Cu, and A*β*-Zn and thus reduced their toxicity.	[2]

SK-N-SH cells	IL-1*β* stimulated	Reduced PGE_2_ and PGD2 production via the reduction of COX-2 activity.	[53]

SK-N-BE cells	TAT-*α*-syn (A30P) and A*β*42 treatment	Inhibited the toxicity induced by TAT-*α*-syn (A30P) and A*β*42 and SIRT1-independently reduced A*β*42 toxicity.	[54]

APP-HEK293 and APP-N2a cell	Treated with A*β*	Played a SIRT1-independent neuroprotective role by activating AMPK.	[55]
